# Innovative synthesis of Sb_2_O_3_@Ag core–shell nanoparticles by two-step laser ablation in liquid for photodetector applications

**DOI:** 10.1039/d6ra07156b

**Published:** 2026-09-25

**Authors:** Mayyadah H. Mohsin, Raid A. Ismail, Ethar Yahya Salih, Mays S. Tareq, Hadeel F. Abbas

**Affiliations:** a Department of Applied Science, University of Technology-Iraq Baghdad Iraq raidismail@yahoo.com; b College of Energy and Environmental Science, Al-Karkh University of Science Baghdad 10081 Iraq ethar988@gmail.com ethar@kus.edu.iq

## Abstract

In this study, a novel metal–semiconductor core–shell nanostructure (Sb_2_O_3_@Ag) was prepared *via* a facile two-step pulsed laser ablation in liquid (PLAL) technique considering different laser fluences. The microstructural analysis revealed a crystalline orthorhombic Sb_2_O_3_ structure, along with a face-centered-cubic phase for Ag nanoparticles; a reduction in the optical band gap from 3.05 eV to 2.82 eV was achieved when the laser fluence was decreased from 6.37 J cm^−2^ to 1.27 J cm^−2^, respectively. The corresponding photodetector, attained at 6.37 J cm^−2^, demonstrated a responsivity value of 113 mA W^−1^, along with a specific detectivity of 3.29 × 10^10^ Jones; these values were attained at an incident light intensity of 2 mW cm^−2^. In contrast, the addressed figures of merit decreased as the incident light intensity increased, with values reaching 22.9 mA W^−1^ and 6.63 × 10^9^ Jones at 36 mW cm^−2^ (*R*_*λ*_ ∝ *P*^−1^). The optimum device delivered rapid and sharp time-resolved characteristics with response/recovery times of 0.24/0.39 s, and other devices demonstrated relatively higher response/recovery times. This indicates faster electron injection than recombination for the optimum photodetector.

## Abstract 2

Two-step PLAL-engineered Sb_2_O_3_@Ag core–shell nanostructures enable a highly sensitive and rapid Sb_2_O_3_@Ag/n-Si photodetector.

## Graphical abstract

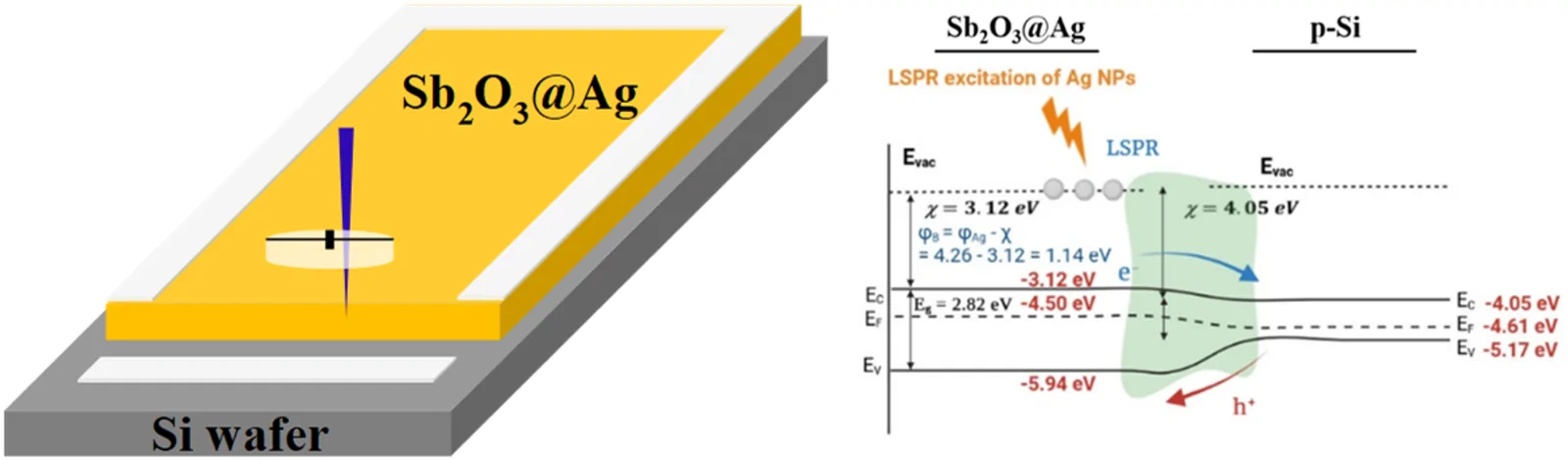

## 1. Introduction

Nanostructured semiconductor materials have facilitated the rapid advancement of optoelectronic devices such as light-emitting diodes (LEDs) and photodetectors.^1–3^ These materials afford enhanced photosensitivity, faster response times, low noise, large linear dynamic regions, and broadband spectral detection capabilities.^4,5^ Silicon-based heterojunctions remain essential components in photodetector technology due to their intrinsic compatibility with metal oxide semiconductors.^6^ Nonetheless, the fundamental limitation of bulk silicon is mainly due to its indirect optical energy gap transitions and significant optical reflectance.^7–9^ Among metal oxide semiconductors, antimony trioxide (Sb_2_O_3_) has drawn significant attention due to its wide optical energy gap, excellent thermal stability, and versatility as both a p-type and an n-type semiconductor.^10^ Nanoscale Sb_2_O_3_ has a relatively large surface-to-volume ratio, which in turn delivers a boosted charge carrier generation ratio. Sb_2_O_3_ plays a crucial role in a number of applications, including energy devices, gas sensors, optical coatings, photodetectors, *etc.*^11,12^ Although Sb_2_O_3_-based optoelectronics, photodetectors in particular, exhibit a sound photoresponsive behavior, the high charge recombination rate of Sb_2_O_3_ restricts the overall performance.^13^ Ag nanoparticles exhibit localized surface plasmon resonance (LSPR) that enhances the interaction between light and matter, which in turn affords an extended optical absorption range to the host matrix.^14^ The coupling effect of Ag and Sb_2_O_3_ increases the efficiency of hot-carrier generation and improves effective photogenerated charge carrier separation at the interface.^15,16^ Many methods, such as chemical vapor deposition,^17^ hydrothermal methods,^18^ vapor condensation,^19^ and pulse laser ablation in liquid (PLAL), are used to synthesize Sb_2_O_3_ nanoparticles.^20,21^ PLAL is one of the most promising routes used to synthesize nanomaterials due to its advantages over other techniques. The main advantages of PLAL include its green protocol, cost-effectiveness, operational simplicity, production of high purity materials, no need for catalyst and vacuum, and size and morphology control.^22–25^ To control the size and morphology of the synthesized nanoparticles, optimizing the laser beam parameters, such as wavelength, pulse duration, pulse repetition frequency, and laser fluence, is necessary. The laser fluence applied in PLAL plays an important role, affecting the formation of plasma and subsequent particle nucleation and growth, directly influencing the physical and functional attributes of the resulting nanoparticles. Despite the documented potential of Ag@Sb_2_O_3_ core–shell nanoparticles attained *via* PLAL, a distinct gap remains in the reported data in regards to the influence of laser fluence parameters on both structural formation and the overall optoelectronic performance, particularly when integrated with an Si substrate as a heterojunction.

In this work, the fabrication process of the Sb_2_O_3_@Ag core–shell/Si heterostructure was well reported considering different laser fluences. Consequently, a systematic optoelectronic investigation was also demonstrated in terms of electrical characterization, figures of merit, and time-resolved performance. Herein, a pronounced photodetection improvement was observed as a function of the applied laser fluence, realizing a direct relation between the tailored core–shell material synthesis and the device performance.

## 2. Experimental

### 2.1. Materials and substrate preparation

A single-side polished p-type Si wafer (Sigma-Aldrich, 99.99% purity, 111) with a 500 µm thickness and an electrical resistivity of 3–5 Ω was used. The wafer was cut into pieces with a size of 1 cm^2^ and then cleaned by ultrasonication in acetone, ethanol, and deionized water (DW) for 15 minutes each, followed by a dip in dilute hydrofluoric acid (HF) to remove the native oxide layer.

### 2.2. Synthesis of Ag@Sb_2_O_3_ core–shell nanoparticles *via* PLAL

**Fig. 1:**
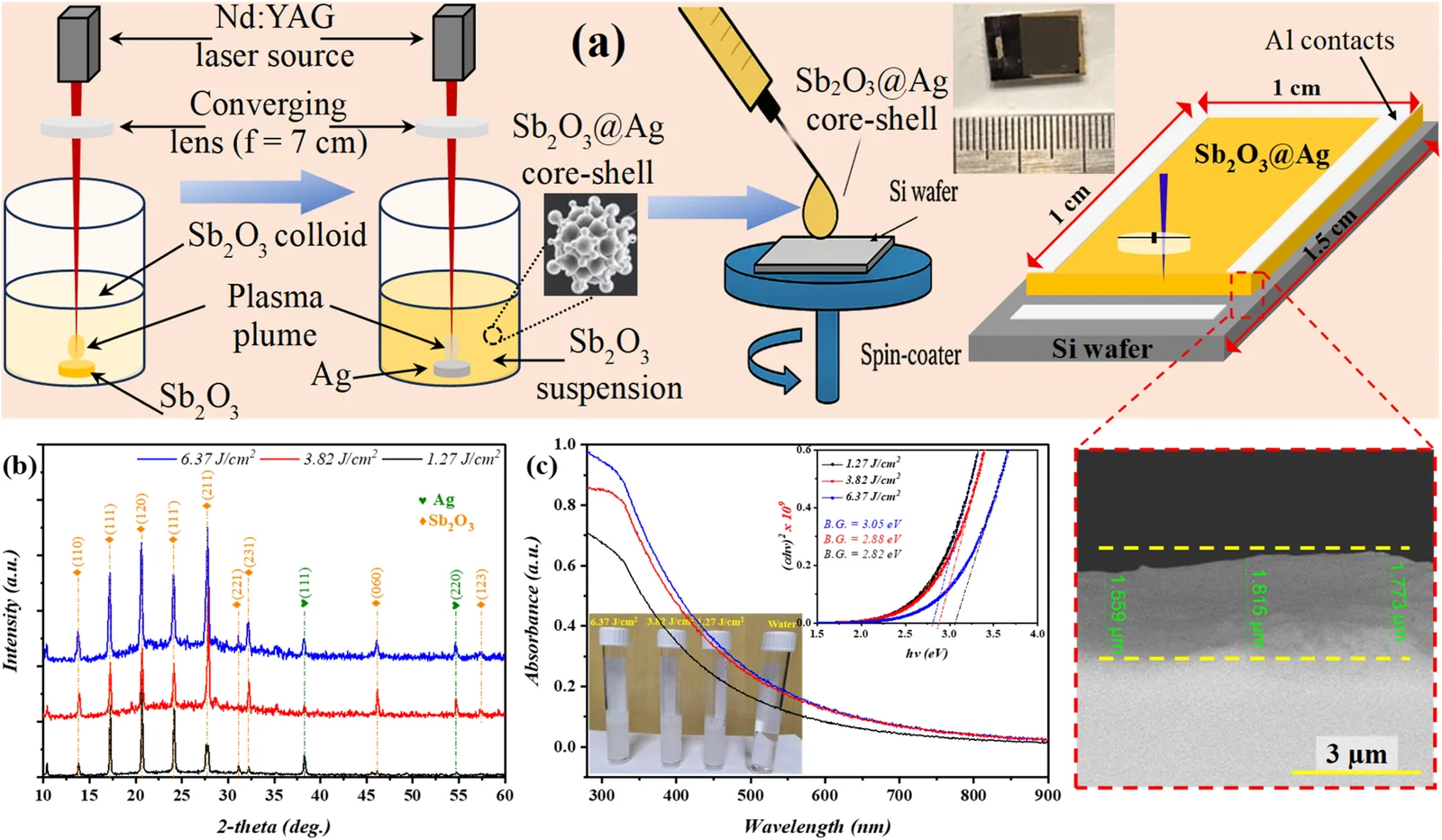
(a) Schematic of the synthesis procedure, (b) XRD patterns and (c) UV-vis spectra of Sb_2_O_3_@Ag colloids prepared by the PLAL technique.

The schematic diagram of the experimental steps for the synthesis of colloidal Sb_2_O_3_@Ag core–shell nanoparticles is shown in Fig. 1a. A high-purity Ag target was immersed in a glass vessel containing DW. A Q-switched Nd:YAG laser (*λ* = 1064 nm, pulse duration = 2 ns, and pulse repetition frequency = 2 Hz) was focused onto the Ag pellet target surface using a converging lens with a 7 cm focal length at a laser fluence of 3.82 J cm^−2^. In the second step, a high-purity Sb_2_O_3_ target was subsequently placed in the preprepared Ag colloid and then irradiated with the same number of laser pulses at three laser fluences (1.27, 3.82, and 6.37 J cm^−2^).

### 2.3. Device fabrication and electrode deposition

The prepared Sb_2_O_3_@Ag colloids were deposited onto cleaned p-Si substrates using a spin-coating technique at 1000 rpm for 20 s; a layer thickness of 1.72 µm was attained, as seen in Fig. 1a. Following the deposition of the Sb_2_O_3_@Ag film, ohmic contacts were made by the evaporation of the in electrode on the Sb_2_O_3_@Ag film and the Al electrode on the back side of the silicon substrate through an interdigitated mask using a thermal evaporation system at a vacuum pressure of 1 × 10^−6^ mbar.

### 2.4. Characterization and optoelectronic measurements

The structural properties of the Sb_2_O_3_@Ag films were examined using an X-ray diffractometer (XRD, Bruker AXS D8). The optical characteristics, including optical absorption, were measured using a UV-vis spectrophotometer (Shimadzu UV-3600). The surface morphology and nanoparticle size distribution were analyzed using a transmission electron microscope (TEM). For device performance evaluation, current–voltage (*I*–*V*) characteristics under dark and illumination conditions were investigated using a source measurement unit (Keithley 2401). The time-dependent photo-response measurements were conducted with histogram-based evaluation (10–90%). The light source was modulated using bandpass filters (340–625 nm range), and the incident power density was calibrated with a precision illuminance meter.

## 3. Results and discussion

Fig. 1b shows the diffraction patterns of Sb_2_O_3_@Ag core–shell nanoparticles synthesized at various laser fluences, where the patterns have a dual-phase feature, indicating that the hybrid architecture is successfully fabricated. The major diffraction peaks corresponding to the (110), (111), (120), (211), and (231) planes are indexed to the orthorhombic phase of the Sb_2_O_3_ shell, which is indexed to JCPDS no. 11-0689. The sharpness and high intensity of these peaks, especially of the (211) reflection, indicate the well-crystalline nature of the host oxide matrix. Additionally, the XRD patterns reveal the existence of the crystalline Ag phase. Specifically, the reflection observed at around 38.2° is associated with metallic FCC Ag, while the peak observed at 2*θ* ≈ 54.5° is assigned to the cubic structure of Ag_2_O (220). As the laser fluence increases from 1.27 to 6.37 J cm^−2^, there is a substantial increase in peak intensity, coupled with a decrease in the full width at half-maximum (FWHM). It is observed that the maximum intensity of Sb_2_O_3_ peaks is observed at the highest laser fluence of 6.37 J cm^−2^.

This singularity indicates that a higher laser fluence results in improved growth kinetics within the plasma plume, which leads to higher structural quality and larger crystalline domains.^24^ In accordance with Scherer's equation, a lower FWHM results in a higher average crystallite size, which is attained at a higher fluence in our experiment. Such an observation is mainly due to the higher pressure and temperature attained at 6.37 J cm^−2^, which in turn provide sufficient thermal energy for smaller particles to merge firmly into relatively larger crystalline clusters. The Ag (111) peak intensity significantly increases as the laser fluence increases, which suggests the usefulness of the applied fluence for the effective ablation of the utilized suspension, where more Ag nanoparticles decorate the surface of the utilized suspension (Sb_2_O_3_), supporting the presented core–shell morphology.

The UV-vis absorption spectra (Fig. 1c) exhibit a very broad band in ultraviolet and visible regions. When the laser fluence increases from 1.27 J cm^−2^ to 6.37 J cm^−2^, a pronounced increase in the absolute value of absorbance can be perceived.^26^ This trend can be correlated with the higher rate of material ablation at larger energy densities, introducing a higher concentration of Ag nanoparticles and a denser Sb_2_O_3_ matrix into the colloidal suspension. The wide absorption band in the visible region is also enhanced by the LSPR effect of the incorporated Ag nanoparticles.^27^ The improved visible-light absorption could be due to Ag nanoparticles' plasmonic contribution, which allows light–matter interactions. The value of the direct allowed bandgap (*E*_g_) was precisely calculated using Tauc's equation.^28^ The energy gap of Sb_2_O_3_@Ag core–shell nanoparticles is found to increase from 2.82 to 3.05 eV as the laser fluence increases from 1.27 to 6.37 J cm^−2^, respectively. The reason behind this result is that as the laser fluence increases, the particle size of Sb_2_O_3_@Ag nanoparticles decreases, and quantum confinement becomes stronger, which leads to an increase in the optical energy gap. Further, the optical band gap of Sb_2_O_3_@Ag core–shell nanoparticles is smaller than that of Sb_2_O_3_ because the Ag shell forms an Ag/Sb_2_O_3_ interface with Sb_2_O_3_, modifying the electronic band structure *via* the transfer of electrons from Ag across the interface and narrowing the optical energy gap. Additionally, the Ag shell may produce defect or energy levels within the energy gap of Sb_2_O_3_, leading to lower-energy optical transitions.

**Fig. 2:**
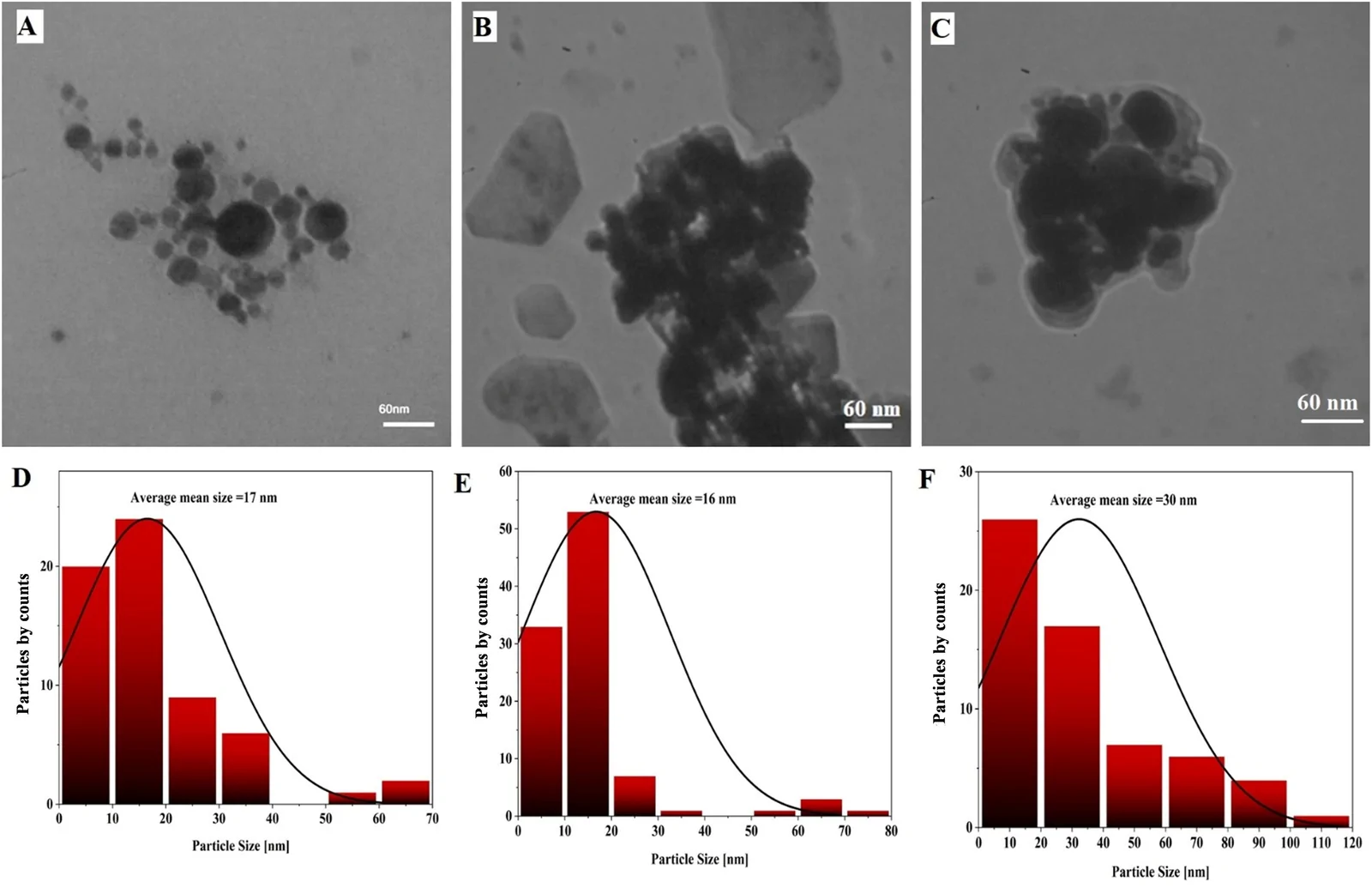
TEM images (A–C) and size distributions (D–F) of Ag NPs, Sb_2_O_3_ NPs, and Sb_2_O_3_@Ag core–shell nanoparticles, respectively.

The TEM image of Ag (Fig. 2A) predominantly exhibits a quasi-spherical morphology with a relatively uniform contrast, while the Sb_2_O_3_ phase appears as irregular, agglomerated clusters with angular or plate-like features (Fig. 2B). The reason behind this finding is the anisotropic crystal growth and low atomic mobility of Sb_2_O_3_ NPs.^29,30^Fig. 2C confirms the formation of Sb_2_O_3_@Ag core–shell nanoparticles because the Sb_2_O_3_ shell covers the core Ag nanoparticles. The average shell thickness was determined using particle size distribution histograms (Fig. 2D–F), which indicate that the hybrid nanostructures span a broad size range (10–80 nm), with an average size of 17 nm, depending on the region analyzed. The smaller nanoparticles (10–30 nm) are primarily because of the formation of Ag nanoparticles through quick condensation in the liquid medium, whereas the less uniform and larger nanostructures (40–80 nm) correspond to Sb_2_O_3_ aggregates arising from higher surface energy and stronger interparticle interactions typical of metal oxides. The observed partial aggregation is expected in surfactant-free laser ablation systems and is primarily governed by van der Waals forces; however, the preservation of distinguishable particle boundaries suggests limited coalescence and retention of nanoscale features.^31–34^

From an optical standpoint, the hybrid Ag/Sb_2_O_3_ system exhibits synergistic behavior. The Ag nanoparticles support strong LSPR in the visible region, leading to enhanced electromagnetic fields on the nanoparticle surface and improved light absorption. In contrast, Sb_2_O_3_, as a wide-bandgap (∼3.6 eV) p-type semiconductor, contributes mainly to UV-region activity and light scattering. The polydispersity of the hybrid system broadens the overall absorption spectrum, enabling extended photo-response across both UV and visible regions. Smaller Ag NPs (<20 nm) facilitate efficient charge transfer due to their high surface area, while larger Sb_2_O_3_ clusters enhance light trapping *via* multiple scattering effects. At the interface with n-type silicon, the hybrid nanostructure introduces two key mechanisms: (I) possible plasmon-assisted charge transfer from Ag NPs across the Ag/n-Si Schottky barrier and (II) the formation of a p–n heterojunction between Sb_2_O_3_ and p-Si. The coexistence of these two interfacial phenomena significantly enhances carrier separation efficiency.^35^ The internal electric field at the Sb_2_O_3_/p-Si junction promotes the directional transport of holes, while the plasmonic excitation in Ag increases the generation of energetic electrons that can overcome the Schottky barrier. This dual mechanism leads to improved photocurrent generation and enhanced photodetector responsivity. However, the TEM observations also indicate that the Sb_2_O_3_ component tends to form agglomerated structures, which may introduce grain boundaries and localized trap states. These defects can act as recombination centers, potentially limiting carrier mobility if not controlled. On the other hand, the clean synthesis environment of laser ablation minimizes chemical impurities, resulting in relatively defect-free interfaces compared to chemically synthesized counterparts. Therefore, optimizing the dispersion and crystallinity of the Sb_2_O_3_ phase remains critical to fully exploit the advantages of the hybrid system.^36^

**Fig. 3:**
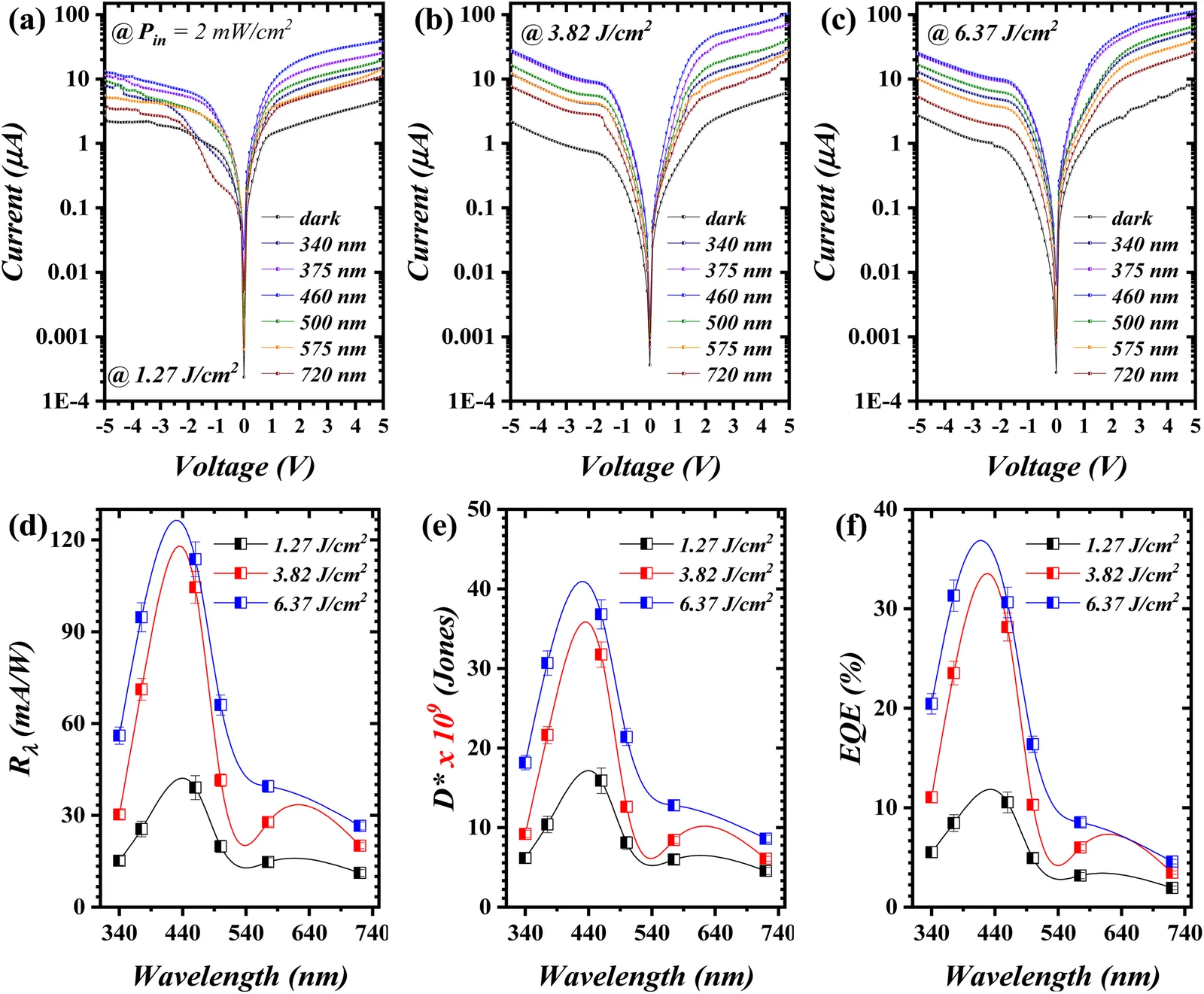
Wavelength dependency profiles: (a–c) current–voltage characteristics under different laser fluences, (d) *R*_*λ*_, (e) D*, and (f) EQE.

The logarithmic *I*–*V* characteristics in the dark and under illumination conditions for Sb_2_O_3_@Ag/Si core–shell heterojunctions fabricated at various laser fluences are shown in Fig. 3a–c. All devices display clear rectifying properties, indicating that a heterojunction diode is formed between the plasmonic Ag@Sb_2_O_3_ nanocomposite and the p-Si substrate. The best rectification factor is observed for the heterojunction prepared at 3.82 J cm^−2^ due to the minimum interfacial defects formed at the interface. Under illumination conditions at various light wavelengths in the forward and reverse directions, the photocurrent of the photodetector increases due to the generation of e–h pairs in the depletion region, and the built-in potential prevents the recombination of the photogenerated charge carriers.^37^ The sample fabricated at a laser fluence of 6.37 J cm^−2^ (Fig. 3c) exhibits the largest photosensitivity 
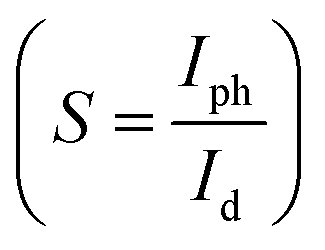
. This is ascribed to the enhanced crystallinity and optimized density of Ag nanoparticles at higher energy densities, enabling the efficient generation and transportation of charge carriers. The photo-response of the Sb_2_O_3_@Ag/p-Si heterojunction was systematically studied in the dark and under monochromatic light excitation from 340 to 720 nm. The highest photocurrent is observed at a wavelength of 460 nm due to absorption at this wavelength in the depletion region as this wavelength is longer than the absorption edge of Sb_2_O_3_@Ag core–shell nanoparticles. The short wavelengths are absorbed at the surface of Sb_2_O_3_@Ag, and due to the presence of dangling bonds, their photocurrent is smaller than that at 460 nm. The responsivity 
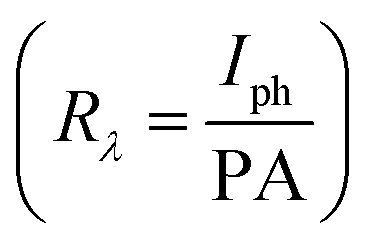
 exhibits two response peaks (Fig. 3d); the first peak at 450 nm belongs to the absorption by the Ag shell, and the second peak is at 580 nm, which comes from the absorption by the core Sb_2_O_3_ NPs. The maximum responsivity is 113 mA W^−1^ at 450 nm for the Sb_2_O_3_@Ag/Si photodetector fabricated at a laser fluence of 6.37 J cm^−2^. This finding can be attributed to the large photocurrent that arises from the large surface area and light trapping. The specific detectivity 
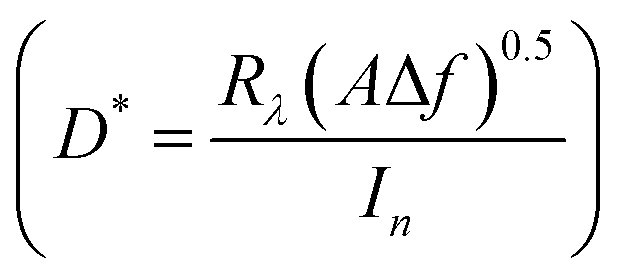
 of the photodetector reaches the maximum value at 450 nm for all photodetectors and then decreases due to recombination (Fig. 3e). The values of D* are 1.59, 3.17, and 3.68 × 10^10^ Jones for Sb_2_O_3_@Ag/Si photodetectors fabricated at laser fluences of 1.27 J cm^−2^, 3.82 J cm^−2^, and 6.37 J cm^−2^, respectively. The high value of D* for the photodetector fabricated at 6.37 J cm^−2^ confirms that it can sense very weak optical signals with respect to the noise level, which makes it suitable for low-light detection.^23,38^ The variation in the D* and NEP of the photodetector with laser fluence can be ascribed to the dependence of the electrical and optical properties of Sb_2_O_3_@Ag core–shell nanoparticles on the laser fluence. The surface area and width of the depletion region are the main parameters affecting the performance of the photodetector. The energy gap and surface area of the photodetector fabricated at a laser fluence of 6.37 J cm^−2^ are larger than those of other photodetectors. Furthermore, the external quantum efficiency 
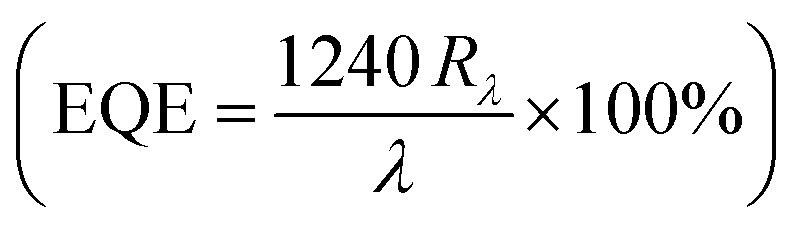
 is found to be in the range of 10–37% at 450 nm (Fig. 3f). The highest value of EQE is obtained for the photodetector fabricated at 6.37 J cm^−2^ due to the synergistic effect of the Sb_2_O_3_ matrix and plasmonic Ag NPs, which significantly decreases the carrier recombination rate.^39^ At this laser fluence, the Ag nanoparticles are at their optimum size for resonantly interacting with the 440 nm excitation light. Such a plasmonic excitation can allow photo-induced charge transfer at the interface, which in turn contributes to the photo-response enhancement.^26,28,39^ Consequently, such carriers are efficiently collected across the p-Si interface by the internal built-in potential so that *R*_*λ*_ and EQE are high.^24,26^

**Fig. 4:**
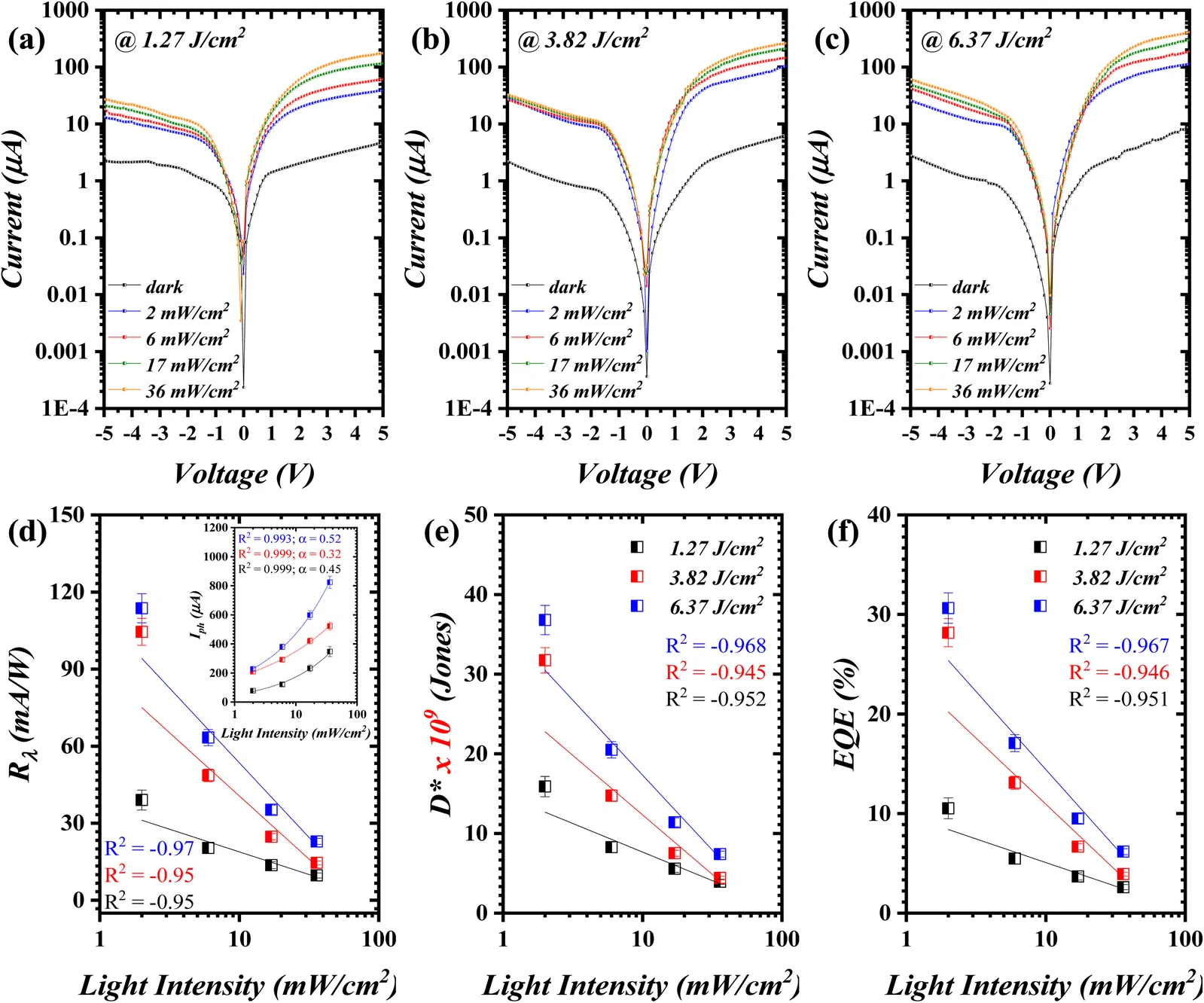
Light intensity dependency profiles: (a–c) current–voltage characteristics under different laser fluences, (d) *R*_*λ*_, (e) D*, and (f) EQE.

Fig. 4a–c presents the *I*–*V* characteristics at different laser fluences, showing an increase in photocurrent (*I*_ph_) with light intensity because the electron–hole pairs are generated more efficiently in the depletion region. In particular, power-law fitting (inset in Fig. 4d) reveals *α* values of 0.52, 0.32, and 0.45 (*R*^2^ = 0.993, 0.999, and 0.999) for laser fluences of 6.37, 3.82, and 1.27 J cm^−2^, respectively; this, in turn, confirms a pronounced sublinear photocurrent dependence on the incident optical power. The effect of the incident light intensity on the *R*_*λ*_, D*, and EQE of the photodetectors is shown in Fig. 4, where a significant reduction in *R*_*λ*_, D*, and EQE (Fig. 4d–f) with increasing light intensity can be observed. The attained sublinear photocurrent dependency (*I*_ph_ ∝ *P*^*α*^,*α* < 1) suggests a power-dependent carrier-loss procedure; this in turn might involve the filling of trap states and improved carrier recombination. The attained outcomes indicate a direct correlation between the enhanced photocurrent and the reduction in the overall photodetector performance as the incident light intensity increases. Additionally, this suggests a pronounced heterojunction functionality under intense optical excitation.^24,40,41^

**Fig. 5:**
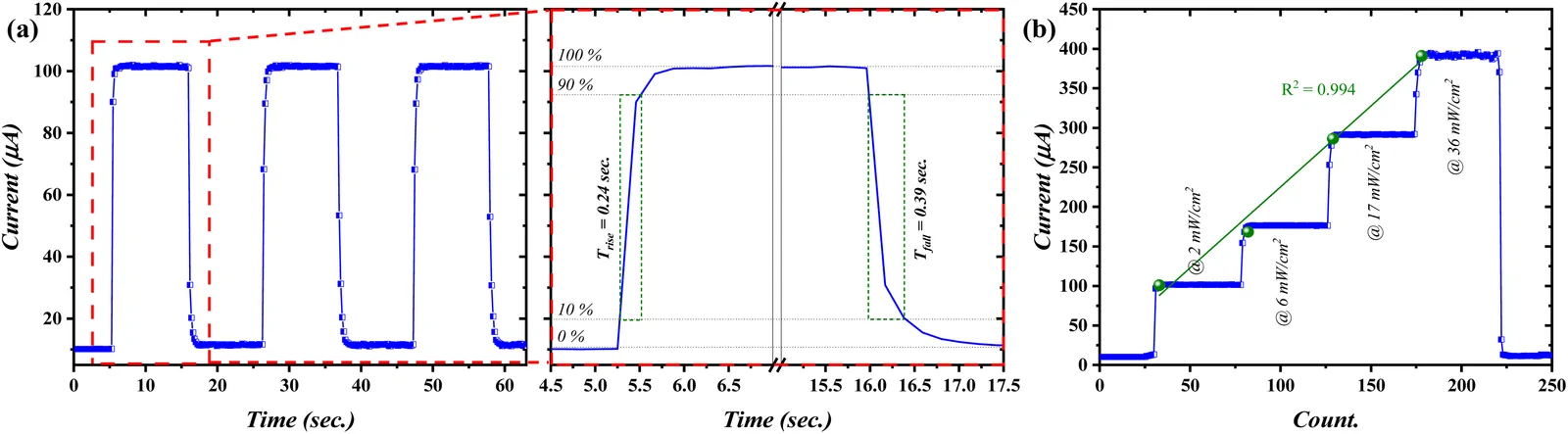
Time-resolved characteristics of the photodetector fabricated at a 6.37 J cm^−2^ laser fluence: (a) switching behavior and (b) time-based power dependency.

The transient photo-response (*I*–*t*) plot of the Al/Sb_2_O_3_@Ag/n-Si photodetector prepared at a 6.37 J cm^−2^ laser fluence is shown in Fig. 5a and that of other devices is presented in S1 and S2. The plot of time-dependent current against the “on–off” ratio is obtained with a sharp transition due to the reproducible switch characteristics. This high repeatability occurring over many cycles verifies the excellent working stability and good cyclic capability under this optical power. The negligible baseline shift or deterioration of photocurrent also indicates efficient carrier extraction and a low trap density in the active regions. These Ag nanoparticles play a vital role in the effective separation of electron–hole pairs. The existence of Ag particles could facilitate the interfacial charge transfer phenomenon *via* the plasmon-assisted procedure, which in turn contributes to the photo-response enhancement. This cooperative mechanism decreases recombination and, therefore, contributes to the higher responsivity of the sample fabricated at an optimum fluence of 6.37 J cm^−2^. A systematic investigation of the laser fluence reveals that 6.37 J cm^−2^ is the optimized value for achieving high-quality structural crystallinity, an efficient bandgap (3.05 eV) for visible light detection and a high-performance p-type Si photodetector device. The extracted integration times (*T*_rise_ and *T*_fall_) of 0.24 s for the rising edge and 0.29 s for the falling edge can be interpreted as characteristic reaction time constants (inset in Fig. 5a). The recovery time is found to be relatively higher than the response time, which indicates a higher rate of carrier recombination processes; this could result from deep-level defects and interfacial states at the fabricated heterostructured interface.^40,42^ Furthermore, the dynamic behavior and photometric linearity of the fabricated device (Al/Sb_2_O_3_@Ag/n-Si at 6.37 J cm^−2^) are shown in Fig. 5b, and those of other devices are presented in S1 and S2. The close-to-unity Pearson correlation (*R*^2^ ≈ 1) indicates a positive correlation between the incident light intensity and the output photocurrent. The attained linear phenomenon indicates the absence of gain saturation, which allows the photogenerated carrier density to be below the trap-state threshold.^40^ Additionally, the unswerving slope in the applied light intensity range indicates rather stable efficiency, implying an unaffected depletion width and band alignment by photon density alteration. This demonstrates efficient carrier extraction through the well-structured heterojunction interface.^40,42^ The measured performance is consistent with the optimized double-heterojunction structure, in which built-in electric fields facilitate efficient carrier separation and, at the same time, minimize recombination-induced nonlinearities.^40,42^

**Fig. 6:**
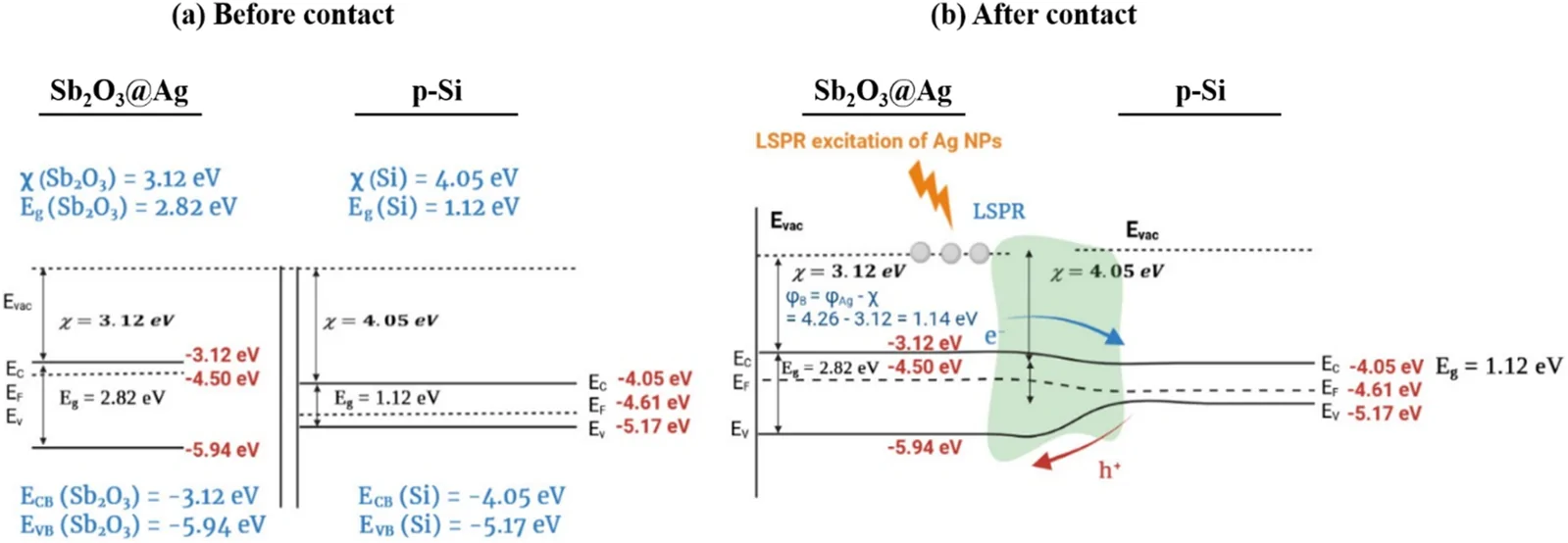
Energy-band diagram of the Sb_2_O_3_@Ag/p-Si heterostructure: (a) before contact and (b) after contact.

The energy band diagrams under illumination of the Sb_2_O_3_@Ag/p-Si heterojunction photodetector before and after contact are shown in Fig. (6). Before contact, the configuration shows an offset between the conduction band edge and valence band edge of Sb_2_O_3_@Ag and those of p-type Si. During heterojunction formation, charge redistribution occurs at the interface to establish Fermi level equilibrium, leading to band bending and the development of an intrinsic electric field. According to the proposed interfacial charge transfer mechanism, the photoexcited electrons in the Sb_2_O_3_@Ag layer are transferred to the lower conduction-band level of p-type Si, and the photogenerated holes are transferred to the Sb_2_O_3_@Ag-Si interface. The spatial distribution of charge carriers reduces the possibility of their recombination and allows effective photocurrent generation in the heterojunction device. The presence of Ag NPs enhances the light absorption through LSPR, which increases the generation and separation of the photogenerated charge carriers produced in the depletion region. The band offsets of the Sb_2_O_3_@Ag/Si photodetector were determined and are found to be Δ*E*_C_ = 0.93 eV and Δ*E*_V_ = 0.77 eV.

## 4. Conclusion

A successful synthesis process for Sb_2_O_3_@Ag core–shell nanoparticles was demonstrated *via* a two-step PLAL technique, along with their microstructural, optical, and photoresponsive features at various laser fluences. The microstructure and optical behavior exhibited a strong correlation with the laser fluence applied, with the optical band gap reaching 3.05 eV at a laser fluence of 6.37 J cm^−2^. At the addressed laser fluence, the fabricated photodetector demonstrated an *R*_*λ*_ of 113 mA W^−1^ at 450 nm, along with a D* of 3.29 × 10^10^ Jones, while the devices attained at 1.27 J cm^−2^ and 3.82 J cm^−2^ delivered D* of 1.59 × 10^10^ and 3.69 × 10^10^ Jones, respectively, suggesting the active role of the utilized laser fluence. The response/recovery times of the optimum device demonstrated values of 0.24/0.39 s, while devices attained at 1.27 J cm^−2^ and 3.82 J cm^−2^ demonstrated values of 0.48/0.60 and 0.27/0.42 s, respectively. These findings establish an operative approach for tailoring device performance through laser fluence alteration for core–shell nanostructured optoelectronic design.

## Conflicts of interest

There are no conflicts to declare.

## Supplementary Material

RA-OLF-D6RA07156B-s001

## Data Availability

All data used are presented in the manuscript and supplementary information (SI). Supplementary information is available. See DOI: https://doi.org/10.1039/d6ra07156b.

## References

[cit1] Nguyen H. P. T., Cùòng T. V., Piao J. (2016). Nanostructured optoelectronics: materials and devices. J. Nanomater..

[cit2] Nallusamy S. (2025). *et al.*, Advances in optoelectronics for environmental and energy sustainability. Next Energy.

[cit3] Abdulhameed M. F., Taha A. A., Ismail R. A. (2021). Improvement of cabbage growth and yield by nanofertilizers and nanoparticles. Environ. Nanotechnol. Monit. Manag..

[cit4] Salih E. Y. (2024). Fabrication and photodetection performance evaluation of nanostructured CdS/Si MSM visible light photodetector. Opt. Mater..

[cit5] Ismail R. A., Hamoudi W. K., Saleh K. K. (2014). Effect of rapid thermal annealing on the characteristics of amorphous carbon/n-type crystalline silicon heterojunction solar cells. cMater. Sci. Semicond. Process..

[cit6] Mishra R. K. , *et al.*, Heterogeneous integration of 2D materials with silicon complementary metal oxide semiconductor (Si-CMOS) devices, in Beyond Si-Based CMOS Devices: Materials to Architecture, Springer. 2024, pp. 149–179

[cit7] Cremades A. (2025). *et al.*, Opportunities of semiconducting oxide nanostructures as advanced luminescent materials in photonics. Adv. Mater..

[cit8] Mhadi R. O., Ismail R. A., Mohsin M. H. (2019). Fabrication of high photosensitivity nanostructured n-Fe2O3/p-Si heterojunction photodetector by rapid thermal oxidation of chemically sprayed FeS2 film. Mater. Res. Express.

[cit9] Noskov A. I. , *et al.*, Overcoming the indirect bandgap: efficient silicon emission via momentum-expanded photonic states. arXiv, 2025, preprint arXiv:2507.19001, 10.48550/arXiv.2507.19001

[cit10] Lee J. H. (2026). *et al.*, Beyond the silicon plateau: a convergence of novel materials for transistor evolution. Nano-Micro Lett..

[cit11] Hadi A. N., Meteab M. H., Mohammed M. K. (2025). Influence of inclusion Sb2O3/NiO nanostructures on the morphological, microstructural, and optical characteristics of PVA polymeric for gamma-ray shielding applications. Rev. Compos. Mater. Av..

[cit12] Rassam N. T. (2022). Synthesis and Characterization of Au/Sb2O3 Ultrathin nanosheet/Au as a High-Performance UV-Photodetector. Polytech. J..

[cit13] He G.-H. (2013). *et al.*, Preparation of novel Sb2O3/WO3 photocatalysts and their activities under visible light irradiation. Mater. Res. Bull..

[cit14] Nanda B. P. (2024). *et al.*, Recent trends and impact of localized surface plasmon resonance (LSPR) and surface-enhanced Raman spectroscopy (SERS) in modern analysis. J. Pharm. Anal..

[cit15] Tan H. (2025). *et al.*, Sb2S3/Sb2O3 Heterojunction for Improving Photoelectrochemical Properties of Sb2S3 Thin Films. Metals.

[cit16] Ma Z. (2019). *et al.*, Chemical vapor deposition growth of high crystallinity Sb2Se3 nanowire with strong anisotropy for near-infrared photodetectors. Small.

[cit17] Khasim S. (2026). *et al.*, Single-crystalline Sb2O3 nanostructures synthesized via chemical vapor deposition for photocatalytic degradation and electrochemical sensing of metronidazole. Catalysts.

[cit18] Edelstein A. S. and Cammaratra R., Nanomaterials: Synthesis, Properties and Applications, CRC press, 1998

[cit19] Zeng D. (2004). *et al.*, Characteristics of Sb2O3 nanoparticles synthesized from antimony by vapor condensation method. Mater. Lett..

[cit20] Kashif M. (2026). *et al.*, Metal oxide nanoparticles: A comprehensive review of recent advances in synthesis strategies, characterization and multifunctional applications. Catalysts.

[cit21] Mendivil M. (2013). *et al.*, Synthesis of silver nanoparticles and antimony oxide nanocrystals by pulsed laser ablation in liquid media. Appl. Phys. A:Mater. Sci. Process..

[cit22] Mohsin M. H., Khashan K. S., Sulaiman G. M. (2023). Optical and structural properties of H-BN@ Gd2O3 nanoflake prepared by laser–induced ablation in liquid. Phys. Scr..

[cit23] Mohsin M. H., Ismail R. A., Mhadi R. O. (2021). Preparation of nanostructured FeS2/Si heterojunction photodetector by laser ablation in water under effect of an external magnetic field: MH Mohsin et al. Appl. Phys. A:Mater. Sci. Process..

[cit24] Shaker S., Ismail R., Ahmed D. (2022). *High-responsivity Bi*
_
*2*
_
*O*
_
*3*
_
*-decorated MWCNT/Si heterojunction photodetector*. J. Inorg. Organomet. Polym..

[cit25] Fadhil F. A., Hussein H. T., Ismail R. A. (2026). Synthesis of PbO nanoparticles/porous Si/c-Si photodetector by one-step laser ablation in liquid. Emergent Mater..

[cit26] Alwazny M. S., Ismail R. A., Salim E. T. (2022). High-quantum efficiency of Au@ LiNbO3 core–shell nano composite as a photodetector by two-step laser ablation in liquid. Appl. Phys. A:Mater. Sci. Process..

[cit27] Naje A. N. (2017). Surface plasmon resonance study of Ag nanoparticles colloidal. Iraqi J. Sci..

[cit28] Naji N. E., Ismail R. A., Aljubouri A. A. (2025). Enhanced responsivity of Fe3O4 nanoparticles/porous silicon heterojunction photodetectors. J. Opt..

[cit29] Abdalaal A. A. (2025). *et al.*, Effect of ablation time on the optical, morphological, and electrical properties of ZnO nanoparticles synthesized via eco-friendly laser ablation in deionized water. J. Mater. Sci.: Mater. Electron..

[cit30] Edan M. S. (2023). *et al.*, Effect of silver nanoparticles synthesized by pulsed laser ablation in liquid on the hematological, hepatic, and renal functions of albino rats. Iraqi J. Sci..

[cit31] Wang H. (2011). *et al.*, A localized surface plasmon resonance light scattering-based sensing of hydroquinone via the formed silver nanoparticles in system. Spectrochim. Acta, Part A.

[cit32] Abbas H. F. (2025). *et al.*, Intense pulsed light annealing of CdS a006Ed Au-decorated CdS nanoparticles for high-performance, self-powered silicon-based heterojunction photodetectors. RSC Adv..

[cit33] Abdulnabi R. K. (2025). *et al.*, High-performance n-V2O5/p-Si heterojunction photodetector prepared by pulsed laser deposition: role of laser fluence. J. Mater. Sci.: Mater. Electron..

[cit34] Mohsin M. H. , Khashan K. S., and Sulaiman G. M., Synthesis of Gd2O3/GdOOH nanocompound using pulsed laser ablation-fragmentation: Controlling experimental parameters for optimal crystallite size distribution, in AIP Conference Proceedings, AIP Publishing LLC, 2025

[cit35] Oshikiri T. (2022). *et al.*, Boosting hydrogen evolution at visible light wavelengths by using a photocathode with modal strong coupling between plasmons and a fabry-pérot nanocavity. Chem. – Eur. J..

[cit36] Mei F. (2019). *et al.*, Recent progress in perovskite-based photodetectors: the design of materials and structures. Adv. Phys.:X.

[cit37] Fu J. (2023). *et al.*, Photodetectors based on graphene–semiconductor hybrid structures: recent progress and future outlook. Adv. Devices Instrum..

[cit38] Gong X. (2009). *et al.*, High-detectivity polymer photodetectors with spectral response from 300 nm to 1450 nm. Science.

[cit39] Abutawahina M. S. (2025). *et al.*, Tailoring ag nanoparticles via PLAL laser energy for High-Performance GaN MSM UV photodetectors. Opt. Mater..

[cit40] Fadhil F. A. (2025). *et al.*, Self-biased TiO2/Si heterostructure for UV-NIR detection via one-step laser ablation process. Mater. Lett..

[cit41] Sze S. M. , Li Y., and Ng K. K., Physics of Semiconductor Devices, John wiley & sons, 2021

[cit42] Salih E. Y. (2025). *et al.*, Fast-response self-powered double-heterojunction n-ZnO/p-ZnTe/n-Si photodetector. Nanoscale Adv..

