# Supplementary material for: Innovative synthesis of Sb2O3@Ag core–shell nanoparticles by two-step laser ablation in liquid for photodetector applications
Source: RSC Adv. 2026 Sep 25;16(45):57686–94. doi: 10.1039/d6ra07156b (PMC13614328; doi:10.1039/d6ra07156b)
Supplement: Supplementary file 1 [file RA-016-D6RA07156B-s001.pdf]

Supplementary Information (SI) for RSC Advances.
This journal is © The Royal Society of Chemistry

Innovative Synthesis of Sb_2O_3@Ag Core-Shell Nanoparticles by Two-step Laser Ablation in Liquid for Photodetector Applications

Mayyadah H. Mohsin[1], Raid A. Ismail[1,*], Ethar Yahya Salih[2,*], Mays S. Tareq[1], Hadeel F. Abbas[1].

[1]Department of Applied Science, University of Technology-Iraq, Baghdad, Iraq.

[2]College of Energy and Environmental Science, Al-Karkh University of Science, Baghdad 10081, Iraq.

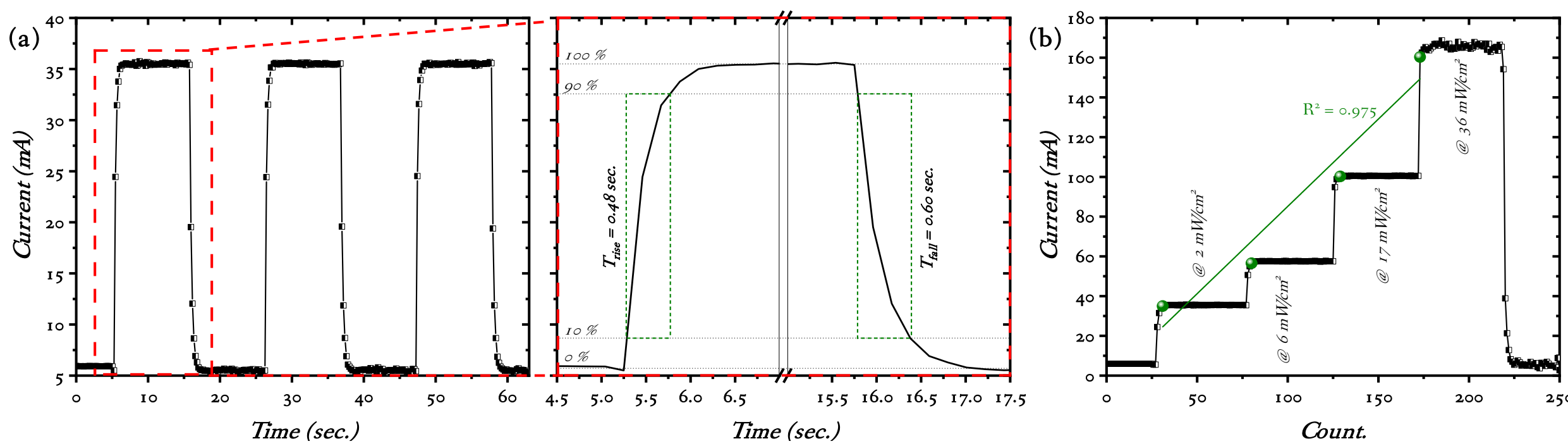

Figure *S1*: Time-resolved characteristics of the fabricated photodetector at 1.27 J/cm² laser fluence; (a) switching behavior and (b) time-based power dependency.

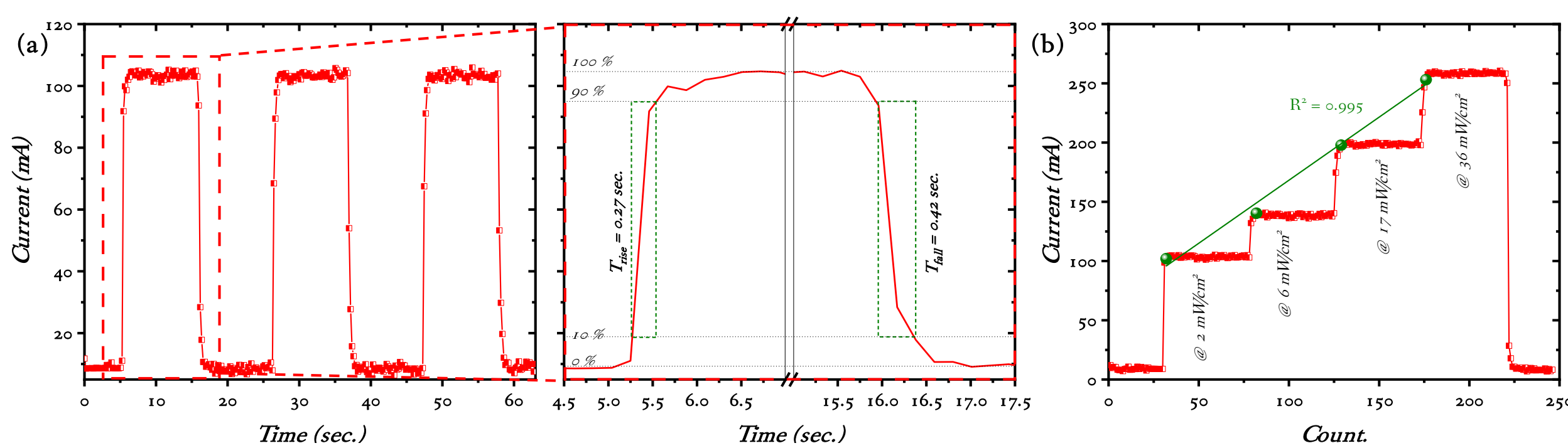

Figure *S2*: Time-resolved characteristics of the fabricated photodetector at 3.82 J/cm² laser fluence; (a) switching behavior and (b) time-based power dependency.

Table *S1*: In-depth key parameters of the fabricated photodetectors at different laser fluence.

Fluence (J/cm²)	$R_{\square}$ (mA/W)	D* (Jones)	EQE (%)	T_{rise}/T_{fall} (sec.)
1.27	39	1.59	10.52	0.48/0.60

3.82	104	3.17	28.17	0.27/0.42
6.37	113	3.68	30.64	0.24/0.39
